# Deformation of the Fermi surface of a spinless two-dimensional electron gas in presence of an anisotropic Coulomb interaction potential

**DOI:** 10.1038/s41598-021-82564-y

**Published:** 2021-02-04

**Authors:** Orion Ciftja

**Affiliations:** Department of Physics, Prairie View A&M University, Prairie View, TX 77446 USA

**Keywords:** Physics, Materials science

## Abstract

We consider the stability of the circular Fermi surface of a two-dimensional electron gas system against an elliptical deformation induced by an anisotropic Coulomb interaction potential. We use the jellium approximation for the neutralizing background and treat the electrons as fully spin-polarized (spinless) particles with a constant isotropic (effective) mass. The anisotropic Coulomb interaction potential considered in this work is inspired from studies of two-dimensional electron gas systems in the quantum Hall regime. We use a Hartree–Fock procedure to obtain analytical results for two special Fermi liquid quantum electronic phases. The first one corresponds to a system with circular Fermi surface while the second one corresponds to a liquid anisotropic phase with a specific elliptical deformation of the Fermi surface that gives rise to the lowest possible potential energy of the system. The results obtained suggest that, for the most general situations, neither of these two Fermi liquid phases represent the lowest energy state of the system within the framework of the family of states considered in this work. The lowest energy phase is one with an optimal elliptical deformation whose specific value is determined by a complex interplay of many factors including the density of the system.

## Introduction

A system of non-interacting electrons, a free electron gas, consists of particles that occupy a range of single-particle quantum states abiding by the rules of Pauli’s exclusion principle^1^ in such a way as to minimize the overall energy. The electrons that occupy the highest energy states are the ones that contribute to nontrivial effects since these electrons can be easily excited to unoccupied states. The highest single-particle energy occupied by electrons in the system at an absolute zero temperature is called the Fermi energy. The surface that the electrons of highest energy occupy in reciprocal space is called the Fermi surface^2^. For the common assumption of free electrons with a given constant isotropic mass, the Fermi surface is circular for a two-dimensional (2D) free electron gas system and spherical for its three-dimensional (3D) counterpart.

For any other model that is more realistic than the free electron gas, one must consider that the electrons move in presence of neutralizing charges (ions) and they do inteact with each other as well as with the neutralizing charge. One of the most popular approaches to treat the neutralizing charge is the use of the jellium approximation. The basic idea behind the approach is to consider the whole system as a uniform gas of electrons moving in a background of uniform positive charge (that approximates the positive ions). The jellium model has been adopted with great success to study the properties of both two-dimensional electron gas (2DEG) and three-dimensional electron gas (3DEG) systems. In addition, electrons under realistic experimental conditions (for instance, in a variety of host semiconductors) may have an effective mass that is different from that of a free electron. The general expectation is that the Fermi surface of an electron gas should be circular or spherical, respectively, in 2D or 3D if the electrons have a constant isotropic (effective) mass and they do not interact with each other. A similar conclusion is expected to be valid for a 2DEG or a 3DEG system in the jellium model approximation if the interaction between electrons involves an isotropic interaction potential (such as the Coulomb interaction) in uniform space^3–9^. The assumption of uniform space means that there no other external potentials affecting the electrons, namely, we are not talking about models where electrons move in a lattice of ions. For example, in the case of electrons with constant isotropic (effective) mass interacting with a standard isotropic Coulomb interaction potential, we have seen very clearly that an elliptical Fermi surface deformation leads to an overall increase of the energy of a spinless 2DEG system^10^.

While an anisotropic (effective) mass^11^ is a trivial source that may cause a deformation of the Fermi surface, in this work we assume that the (effective) mass of the electrons is constant and, thus, our principal concern is to study the effects of a specific anisotropic interaction potential in the overall stability of the system. Therefore, we focus our attention on a 2DEG model in the jellium approximation and consider the possibility of an elliptical deformation of the Fermi surface in 2D reciprocal space induced by an internal anisotropy of the interaction potential. For simplicity, we assume a fully spin-polarized (spinless) system of electrons described by a family of wave functions written as anti-symmetrized Slater determinant wave functions of plane waves^12^ with quantum states that belong to an elliptical domain in 2D reciprocal space.

The model under consideration is that of a 2DEG system of particles with an (effective) constant isotropic mass but interacting with an anisotropic Coulomb interaction potential that has been considered recently in the context of studies of a 2DEG system in the quantum Hall regime^13,14^. The objective of the study is to understand how the interplay of various factors determines the degree of elliptical deformation of the Fermi surface as a function of the density of the system for the given anisotropic interaction potential. The density of the system determines to a large extent whether the total energetic stability of the system is dominated by the kinetic energy (at large density) or potential energy (at low density). Ultimately, it is the non-trivial interplay of several factors (such as degree of anisotropy of the potential, density of the system, etc.) that determines the magnitude of the elliptical deformation of the Fermi surface. From this point of view, the main motivation of this work is to shed light on the nature of novel exotic anisotropic electronic Fermi liquid phases with deformed Fermi surfaces driven by an anisotropic interaction potential between particles (or a combination of an anisotropic potential and an anisotropic effective mass if one chooses to incorporate an anisotropic mass as an additional feature of the model at a later phase). In this light, the ideas of this work may apply and motivate experiments in a 2DEG system confined in modulation-doped AlAs (or similar) quantum wells^15^. Standard experimental techniques applied to such materials allow one to create systems where the electrons possess an anisotropic effective mass. The effective mass anisotropy of such a system combined with the inherent piezo-electric effect of the samples should lead to interesting anisotropic features. An additional effective anisotropic interaction between the particles can be easily induced in such systems by externally tuning the piezo-electric properties of the sample. An anisotropic Coulomb interaction potential of the nature studied here captures these basic physical elements that, overall, are relevant to real physical systems with forms of intrinsic anisotropy as mentioned above. This mindset hints to possible new experiments that can be performed in such 2DEG systems with the idea to tune at least one source of anisotropy (the interaction potential or the anisotropic effective mass) and then look for experimental signatures of transport anisotropy as a function of other controllable factors.

## Theory and model

The 2DEG model under consideration is well known in the literature and has been explained in details in several publications^16–18^. It is assumed that electrons are spinless and possess a constant isotropic (effective) mass, *m*. The system of *N* electrons is embedded in a uniformly charged neutralizing jellium background with area $A=L^2$ that fills the 2D square region:

1 $$\begin{aligned} \Omega : \left\{ -\frac{L}{2} \le x, y \le +\frac{L}{2} \right\} . \end{aligned}$$

The electron number density,

2 $$\begin{aligned} \rho _{0}=\frac{N}{L^2} , \end{aligned}$$

is constant in the thermodynamic limit, $N \rightarrow \infty$ and $L \rightarrow \infty$. The formalism and notation is similar to that for a finite spinless 2DEG system studied in Ref. [16]. Major differences and new added crucial elements consist of: (i) The current model is for an infinite 2DEG system; (ii) The interaction potential is different from a standard isotropic Coulomb potential and (iii) The current model assumes that the Fermi surface of an infinite 2DEG system can be either circular or elliptically deformed^10^. The Hamiltonian of the system is given by

3 $$\begin{aligned} {\hat{H}}={\hat{T}}+{\hat{U}} , \end{aligned}$$

where ${\hat{T}}$ is the usual kinetic energy operator and

4 $$\begin{aligned} {\hat{U}}={\hat{U}}_{ee}+{\hat{U}}_{eb}+{\hat{U}}_{bb} , \end{aligned}$$

is the potential energy operator that incorporates the electron-electron (ee), electron-background (eb) and background-background (bb) interaction potential energies.

A major difference between this model and other models in the literature is the assumption that the charges interact with an anisotropic Coulomb interaction potential as recently considered in the context of quantum Hall regime studies^13,14^. For a pair of two electrons, one can write this anisotropic Coulomb interaction potential as:

5 $$\begin{aligned} v_{\gamma }(\vec {r}_{i} - \vec {r}_{j})= \frac{k_e \, e^2}{\sqrt{ \frac{x_{ij}^2}{\gamma ^2} +\gamma ^2 \, y_{ij}^2 }} , \end{aligned}$$

where $\gamma > 0$ is a phenomenological interaction anisotropy parameter, $\vec {r}_i-\vec {r}_j = \vec {r}_{ij}=(x_{ij}, y_{ij})$ is the vector that separates the positions of particles *i* and *j*, $-e$ $(e>0)$ is electron’s charge and $k_e$ is Coulomb’s electric constant. The standard isotropic Coulomb interaction potential is recovered for $\gamma =1$. One can calculate quite generally that, for the same magnitude of separation between two electrons, one has:

6 $$\begin{aligned} \frac{v_{\gamma }(x_{ij}=d,y_{ij}=0)}{v_{\gamma }(x_{ij}=0,y_{ij}=d)}=\gamma ^2. \end{aligned}$$

This means that, for these conditions, the repulsion along the *x* direction is stronger than the repulsion along the *y*-direction if $\gamma > 1$. Obviously, our research interest is only on the $\gamma \ne 1$ anisotropic case.

Similarly to the case of a system of *N* electrons interacting with an isotropic Coulomb interaction potential one can write:

7 $$\begin{aligned} {\hat{U}}_{ee} = \frac{1}{2} \sum _{i=1}^{N} \sum _{j \ne i}^{N} {v}_{\gamma }( \vec {r}_{i}-\vec {r}_j ) ,\end{aligned}$$

8 $$\begin{aligned} {\hat{U}}_{eb} = -\rho _0 \sum _{i=1}^{N} \int _{\Omega } d^{2}r^{\, \prime } \, {v}_{\gamma }( \vec {r}_i-\vec {r}^{\, \prime } ) \ , \end{aligned}$$

and

9 $$\begin{aligned} {\hat{U}}_{bb}=\frac{\rho _0^2}{2} \int _{\Omega } d^2r \int _{\Omega } d^2r^{\prime } \, \, {v}_{\gamma }( \vec {r}-\vec {r}^{\, \prime }) \ , \end{aligned}$$

where the usual Coulomb potential has now been replaced by its anisotropic $\gamma$-dependent counterpart and $\Omega$ is the 2D square region given by Eq. (1).

We describe the ground state of the spinless 2DEG system by a family of normalized Slater determinant wave functions of 2D plane waves, $\phi _{\vec {k}}(\vec {r})=e^{i \, \vec {k} \, \vec {r}}/\sqrt{A}$ (periodic boundary conditions are imposed):

10 $$\begin{aligned} \Psi (\alpha )=\frac{1}{\sqrt{N!}} \, Det \left\{ \phi _{\vec {k}_1}(\vec {r}_1), \ldots , \phi _{\vec {k}_N}(\vec {r}_N) \right\} , \end{aligned}$$

where the set of occupied plane wave $\vec {k}$-states fills a reciprocal space region/domain with elliptical Fermi surface:

11 $$\begin{aligned} D_{\vec {k}}: \left\{ \frac{k_x^2}{k_a^2}+\frac{k_y^2}{k_b^2} \le 1 \right\} . \end{aligned}$$

The parameter $\alpha > 0$ that appears as argument of the wave function $\Psi (\alpha )$ in Eq. (10) is defined as:

12 $$\begin{aligned} \alpha =\frac{k_{a}}{k_F}=\frac{k_F}{k_{b}} , \end{aligned}$$

where $k_{F}$ represents the value of the Fermi wave number for a circular Fermi surface. The parameter, $\alpha$ describes the amount of the elliptic deformation of the Fermi surface. The choice in Eq. (12) guarantees the condition that:

13 $$\begin{aligned} k_a \, k_b = k_F^2=4 \, \pi \, \rho _0 . \end{aligned}$$

Based on the notation adopted, a wave function of the form $\Psi (\alpha =1)$ represents a Fermi liquid state with circular Fermi surface. On the other hand, any wave function $\Psi (\alpha \ne 1)$ represents a state with elliptical Fermi surface.

The total kinetic energy of the system (more precisily, the expectation value of the operator ${\hat{T}}$) is written as:

14 $$\begin{aligned} T(\alpha )=\sum _{ \{ D_{\vec {k}} \}} \frac{\hbar ^2 \, |\vec {k}|^2}{2\, m} , \end{aligned}$$

where the sum is over all $\vec {k}$-states in the elliptical region $D_{\vec {k}}$ given by Eq. (11). The total potential energy of the system (namely, the expectation value of the operator ${\hat{U}} = {\hat{U}}_{ee} + {\hat{U}}_{eb} + {\hat{U}}_{bb}$) can be written as:

15 $$\begin{aligned} U(\alpha ,\gamma )= -\frac{1}{2} \, \int _{\Omega } d^2r_1 \int _{\Omega } d^2r_2 \, |\rho (\alpha ,\vec {r}_1,\vec {r}_2)|^2 \, v_{\gamma }(\vec {r}_{2}-\vec {r}_{1}) , \end{aligned}$$

where $\Omega : \left\{ -\frac{L}{2} \le x, y \le +\frac{L}{2} \right\}$ represents the square 2D region in the thermodynamic limit of $L \rightarrow \infty$. The quantity $\rho (\alpha ,\vec {r}_1,\vec {r}_2)$ which appears in Eq. (15) is the one-particle density matrix:

16 $$\begin{aligned} \rho (\alpha ,\vec {r}_1,\vec {r}_2)= \sum _{ \{ D_{\vec {k}} \}} \phi _{\vec {k}}(\vec {r}_1)^{*} \, \phi _{\vec {k}}(\vec {r}_{2})= \frac{1}{A} \, \sum _{ \{ D_{\vec {k}} \}} e^{ i \, \vec {k} \, (\vec {r}_2-\vec {r}_1)} , \end{aligned}$$

where the asterisk ($^{*}$) means complex conjugation.

Note that the total kinetic energy $T(\alpha )$ depends only on the elliptical deformation parameter of the Fermi surface, $\alpha$ but not on the nature of the interaction potential between particles. On the other hand, the total (exchange) potential energy $U(\alpha ,\gamma )$ depends on both parameters, $\alpha$ and $\gamma$. In the thermodynamic limit the sum over $\vec {k}$-states transforms into an integral over an elliptical Fermi surface in 2D reciprocal space. As soon as the expectation value of the Hamiltonian is calculated with respect to the free fermion trial ground state wave function, the used approach is in fact, a Hartree–Fock (HF) approximation. It is well known that, while being useful and versatile, the HF approximation has its weaknesses since the so-called correlation energy is missing in the calculations. The difference between the unknown exact energy and the sum of non-interacting kinetic energy and the exchange energy is commonly referred to as correlation energy in condensed matter physics. In a HF treatment, one can view the HF energy terms as coming from first-order perturbation theory while correlation energy comes from all other terms in the perturbation expansion. As a result, the neglect of the correlation energy limits the accuracy of the HF method. The calculation of the correlation energy is a very difficult task. It has been a major activity for many decades and generally leads to a problem that can be handled only via numerical treatments. A major technical breakthrough to improve the accuracy of the results has been the implementation of numerical quantum Monte Carlo (QMC) methods few decades ago^3^. Such methods can yield accurate values for the ground-state energies of an electron gas and its phase diagram (where the typical competing phases considered are the spin unpolarized paramagnetic liquid phase, the spin polarized ferromagnetic liquid phase and the Wigner crystal state). From this standpoint, the HF results presented in this work should not be expected to be as quantitatively accurate as the results obtained via robust QMC methods. However, despite the shortcomings, the HF method works reasonably well for various systems and may lead to qualitatively accurate results and predictions that can be checked for accuracy at a later stage. For instance, as pointed out by Bloch^19^ long time ago, the HF approximation was able to predict correctly (in a qualitative sense), the existence of a ferromagnetic spin-polarized liquid phase of a free electron gas. Stabilization of such a phase for a given range of densities has been confirmed in several QMC studies^3,5,7,8,20^. By the same token, one must recognize that the best HF ground state is by no means obvious. Even within the HF approximation there are still more complicated choices of one-electron orbitals that can affect the results. Therefore, one should be cautious when judging various predictions.

This being said, we take the opportunity to emphasise the point that the key objective of the subsequent calculations is to obtain general results that apply to arbitrary values of the two parameters, $\alpha$ and $\gamma$. The technical calculation of the kinetic and potential energy terms follows the same formalism as that adopted for a standard isotropic Coulomb interaction potential^10^ but with the additional twist of having to deal with a more general $\gamma$-dependent anisotropic potential. In order to achieve a better readability and reproducibility of the results we provide details of the calculations and the formalism in several appendices rather than simply citing previous work.

## Results and discussions

The kinetic energy per particle, $t(\alpha )=T(\alpha )/N$ corresponding to a state with a given elliptical deformation of the Fermi surface (namely, for a given value of $\alpha$) is calculated in Appendix A. The final result is:

17 $$\begin{aligned} t(\alpha )= \frac{\epsilon _F}{4} \left( \alpha ^2+\frac{1}{\alpha ^2} \right) \end{aligned}$$

where

18 $$\begin{aligned} \epsilon _F=\frac{\hbar ^2 \, k_F^2}{2 \, m} , \end{aligned}$$

is the Fermi energy for a spinless 2DEG system with a circular Fermi surface. The kinetic energy per particle does not depend on the interaction potential, namely, in our case does not depend on the value of the anisotropic interaction parameter, $\gamma$.

On the other hand, the potential energy per particle, $u(\alpha ,\gamma )=U(\alpha ,\gamma )/N$ is a function of both parameters $\alpha$ and $\gamma$. The calculation of the potential energy in Eq. (15) relies on the expression for $\rho (\alpha ,\vec {r}_1,\vec {r}_2)$ that is obtained in Appendix B. The details of the calculation for the potential energy per particle are provided in Appendix C. The final result for the potential energy per particle is shown below:

19 $$\begin{aligned} u(\alpha , \gamma )= -\frac{8}{3 \, \pi ^2} \, k_F \, k_e \, e^2 \, F(\alpha \, \gamma ) , \end{aligned}$$

where

20 $$\begin{aligned} F(x)= \frac{1}{x} K\left( m=1-\frac{1}{x^4} \right) , \end{aligned}$$

and *K*(*m*) is a complete elliptic integral of the first kind^21^. Obviously, it is understood from the context that the argument *m* of the complete elliptic integral function represents a dummy parameter that has nothing to do with the mass of the electrons. Note the following special result:

21 $$\begin{aligned} F(x=1)=\frac{\pi }{2}. \end{aligned}$$

It can be easily checked that *F*(*x*) has a maximum at $x=1$ (which implies that its negative, $-F(x)$ has a minimum at $x=1$). Recall that the potential energy per particle is proportional to $-F(x)$. These results imply that the minimum potential energy for particle is obtained for an elliptical Fermi surface deformation that corresponds to a parameter $\alpha =1/\gamma$ and results in an energy of:

22 $$\begin{aligned} u \left( \alpha =\frac{1}{\gamma }, \gamma \right) = -\frac{4}{3 \, \pi } \, k_F \, k_e \, e^2 . \end{aligned}$$

The two quantum states, $\Psi (\alpha =1)$ and $\Psi (\alpha =\frac{1}{\gamma })$ are special. The first one represents a Fermi liquid state that minimizes the kinetic energy while the second one is the state that minimizes the potential energy.

We remind the reader that a value of $\alpha =1$ represents a circular Fermi surface while a value of $\gamma =1$ corresponds to a standard isotropic Coulomb potential. The kinetic energy per particle and the potential energy per particle for the case of a spinless 2DEG with circular Fermi surface and standard isotropic Coulomb potential ($\gamma =1$) respectively read:

23 $$\begin{aligned} t(\alpha =1)=\frac{\epsilon _F}{2} \ \ \ ; \ \ \ u(\alpha =1,\gamma =1)= -\frac{4}{3 \pi } \, k_F \, k_e \, e^2. \end{aligned}$$

The Fermi energy for a circular Fermi surface can be written as:

24 $$\begin{aligned} \epsilon _F=\frac{\hbar ^2 \, k_F^2}{2 \, m}= (k_F \, a_B)^2 \, \frac{k_e \, e^2}{2 \, a_B}, \end{aligned}$$

where

25 $$\begin{aligned} a_B= \frac{\hbar ^2}{k_e \, m \, e^2} , \end{aligned}$$

is the Bohr radius for Hydrogen atom and $k_e \, e^2/(2 \, a_B)=1 \, Ry$ is a commonly used unit of energy. Similarly, one can write:

26 $$\begin{aligned} k_F \, k_e \, e^2= 2 \, (k_F \, a_B) \, \frac{k_e \, e^2}{2 \, a_B} . \end{aligned}$$

With help from Eqs. (24), (25) and (26) one can write the quantities in Eqs. (17) and (19) as:

27 $$\begin{aligned} t(\alpha )= \frac{(k_F \, a_B)^2}{4} \, \left( \alpha ^2+\frac{1}{\alpha ^2} \right) \, \frac{k_e \, e^2}{2 \, a_B} \end{aligned}$$

and

28 $$\begin{aligned} u(\alpha , \gamma )= -\frac{16}{3 \, \pi ^2} \, (k_F \, a_B) \, F(\alpha \, \gamma ) \, \frac{k_e \, e^2}{2 \, a_B} . \end{aligned}$$

One may write the electron number density for a 2DEG as

29 $$\begin{aligned} \rho _{0}= \frac{1}{\pi \left( r_s \, a_B \right) ^2}, \end{aligned}$$

where $r_s$ is the Wigner-Seitz radius. As a result, we can express the kinetic energy per particle solely as a function of parameter $\alpha$ and $r_s$ and, similarly, one can express the potential energy per particle as a function of parameters, $\alpha$, $\gamma$ and $r_s$:

30 $$\begin{aligned} t(\alpha ,r_s)= \frac{1}{r_s^2} \left( \alpha ^2+\frac{1}{\alpha ^2} \right) \, \frac{k_e \, e^2}{2 \, a_B} , \end{aligned}$$

and

31 $$\begin{aligned} u(\alpha , \gamma , r_s)= -\frac{32}{3 \, \pi ^2} \, \frac{1}{r_s} \, F(\alpha \, \gamma ) \, \frac{k_e \, e^2}{2 \, a_B}. \end{aligned}$$

Note the notation in Eqs. (30) and (31) where the kinetic and the potential energy per particle are denoted, respectively, as $t(\alpha ,r_s)$ and $u(\alpha ,\gamma ,r_s)$. The total energy per particle of the system is written as:

32 $$\begin{aligned} \epsilon (\alpha ,\gamma ,r_s)=t(\alpha ,r_s)+u(\alpha ,\gamma ,r_s), \end{aligned}$$

where $\alpha$ measures the degree of elliptical deformation of the Fermi surface, $\gamma$ is the anisotropic Coulomb interaction parameter and $r_s$ gauges the density of the 2DEG system. One has:

33 $$\begin{aligned} \epsilon (\alpha ,\gamma ,r_s)= \Bigg [ \frac{1}{r_s^2} \left( \alpha ^2+\frac{1}{\alpha ^2} \right) -\frac{32}{3 \, \pi ^2} \, \frac{1}{r_s} \, F(\alpha \, \gamma ) \Bigg ] \frac{k_e e^2}{2 \, a_B}, \end{aligned}$$

where *F*(*x*) is given from Eq. (20).

**Figure 1:**
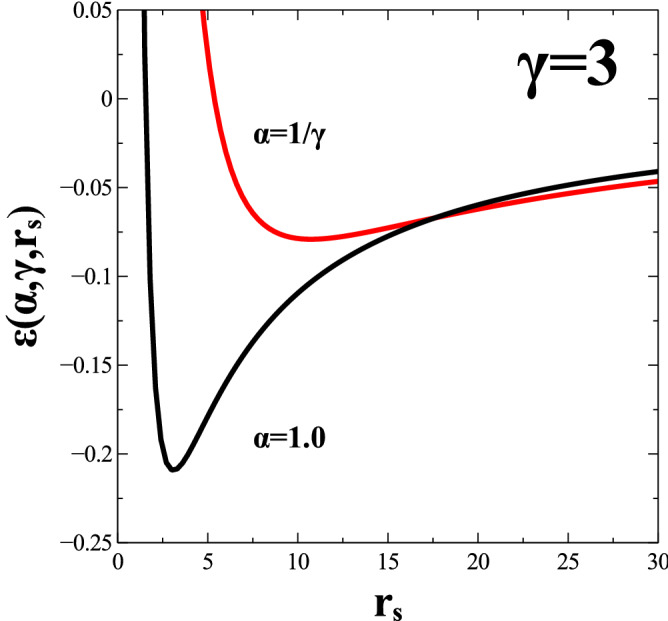
Total energy per particle for the cases of $\alpha =1$ and $\alpha =1/\gamma$, namely, $\epsilon (\alpha =1,\gamma ,r_s)$ and $\epsilon (\alpha =1/\gamma ,\gamma ,r_s)$ as a function of $r_s$ corresponding to a spinless 2DEG system with anisotropic Coulomb interaction potential with $\gamma =3$. The energy is measured in units of $\frac{k_e e^2}{2 \, a_B}$. Note that a 2D Wigner crystal state forms at values $r_s > 34$ (see Pg. 53 of Ref. [22]). This means that the interval of $r_s$ values chosen for the current calculations is suitably restricted to densities where the Wigner lattice is not yet formed.

It has been shown^10^ for the case of an isotropic Coulomb interaction potential ($\gamma =1$) that the state $\Psi (\alpha =1)$ constitutes the lowest energy state at any selected densities (different values of $r_s$). This state represents a spinless 2DEG with circular Fermi surface. Therefore, our main interest is not at all on a standard isotropic Coulomb interaction potential, but rather on an anisotropic one generated by $\gamma \ne 1$ values.

To start with, let us consider an anisotropic Coulomb interaction potential with $\gamma =3$. In Fig. 1 we compare the total energy per particle for the state $\Psi (\alpha =1)$ (the one that gives the lowest kinetic energy) to the state with an elliptical Fermi surface, $\Psi (\alpha =\frac{1}{\gamma })$ (the one that gives the lowest potential energy) for an anisotropic Coulomb interaction potential with value of $\gamma =3$ at various densities (values of $r_s$) of the system. Note the crossing transition at some specific value of $r_s$. Kinetic energy dominates at small $r_s$ and, for these values, $\Psi (\alpha =1)$ has lower energy than the state $\Psi (\alpha =\frac{1}{\gamma })$ that represents the state with the lowest potential energy. However, we remark that as long as $\gamma > 1$, the global energy minimum is neither the state with $\alpha =1$, nor the state $\alpha =1/\gamma$. The global minimum of energy is achieved for some value of $\alpha$ between $1/\gamma$ and 1. The precise value depends on the specific values of $r_s$ and $\gamma$ and can determined numerically.

Having reached this point, we recall that the present treatment does not deal with the Wigner crystal phase of the electrons which necessarily exists when the density of the 2DEG is sufficiently low. At very low densities the true ground state of the electron gas bears no resemblance to the forms described here. In the low density limit the electron gas can be shown to crystalize as a Wigner crystal whose description is outside the scope of this work. Although, the present numerical results formally embrace a wide interval of densities (for instance, with $r_s$ going up to 30 in Fig. 1), we had this situation very much in mind when we performed our calculations. To this effect, we suitably restricted the interval of the Wigner-Seitz radius $r_s$ to values where the Wigner lattice is not yet formed. Although some numerical “fine-tuning” still goes on to determine the precise value of $r_s$ for which the Wigner crystal becomes the stable state, we took the value $r_s > 34$ reported in Pg. 53 of Ref. [22] as the accepted one where the Wigner crystal has lower energy than its 2DEG counterpart. For this reason, we restricted $r_s$ in our calculations to values that are not larger than $r_s=30$.

**Figure 2:**
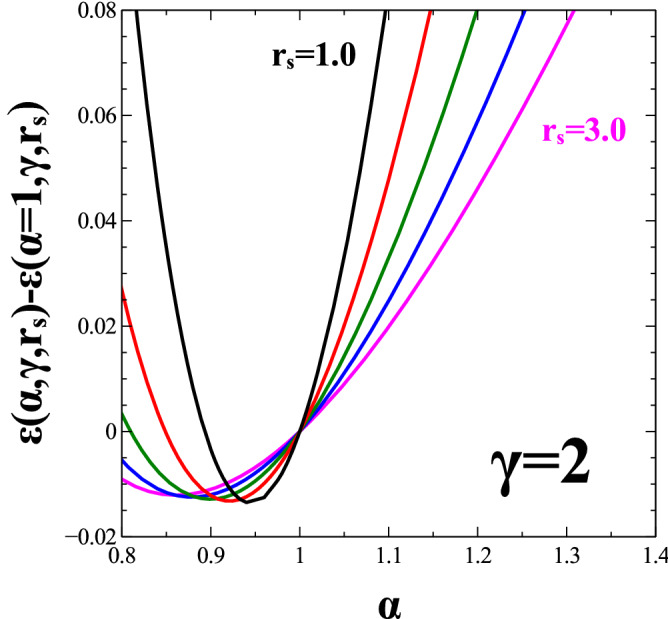
The energy per particle difference, $\epsilon (\alpha ,\gamma ,r_s)-\epsilon (\alpha =1,\gamma ,r_s)$ as a function of $\alpha$ for parameter, $\gamma =2$ for values of $r_s=1.0, 1.5, 2.0, 2.5$ and 3.0 (from left to right). The energy is measured in units of $\frac{k_e e^2}{2 \, a_B}$.

In Fig. 2 we calculate the difference of the energy per particle, $\epsilon (\alpha ,\gamma ,r_s)-\epsilon (\alpha =1,\gamma ,r_s)$ as a function of $\alpha$ for a selected value $\gamma =2$ of the anisotropic Coulomb interaction parameter. Recall that the state $\alpha =1$ represents a spinless 2DEG with circular Fermi surface. To see the influence of the density on the energetic stability of the system we selected five diferent values of $r_s$ ranging from 1.0 (left, top right corner) to 3.0 (right, top right corner). Note that, for $\gamma =2$, the state $\Psi (\alpha =1/2)$ that mimimizes the potential energy is way too high in energy (out of the scale in Fig. 2). However, as pointed out earlier, neither the state $\Psi (\alpha =1)$ that minimizes the kinetic energy is the global minimum. As seen from from Fig. 2 the global energy minimum for a given value of $r_s$ is achieved for some value of $\alpha$ between 0.5 and 1. For the case of $\gamma =2$ and $1 \le r_s \le 3$ shown in Fig. 2 the global minimum is achieved for an $\alpha$ much closer to 1 than to $1/\gamma =0.5$.

Let’s try, now, to understand in more detail the energy competition between the two Fermi liquid phases $\Psi (\alpha =1)$ and $\Psi (\alpha =\frac{1}{\gamma })$ for the case of an anisotropic Coulomb interaction potential with $\gamma \ne 1$ values. The corresponding energies are:

34 $$\begin{aligned} \epsilon (\alpha =1,\gamma ,r_s)= \Bigg [ \frac{2}{r_s^2} -\frac{32}{3 \, \pi ^2} \, \frac{1}{r_s} \, F( \gamma ) \Bigg ] \frac{k_e \, e^2}{2 \, a_B} , \end{aligned}$$

and

35 $$\begin{aligned} \epsilon \left( \alpha =\frac{1}{\gamma },\gamma ,r_s \right) = \Bigg [ \frac{1}{r_s^2} \left( \gamma ^2+\frac{1}{\gamma ^2} \right) -\frac{16}{3 \, \pi } \, \frac{1}{r_s} \Bigg ] \frac{k_e \, e^2}{2 \, a_B}. \end{aligned}$$

For a small anisotropy value of the anisotropic Coulomb interaction potential so that $\gamma \approx 1$ one can expand:

36 $$\begin{aligned} \gamma ^2+\frac{1}{\gamma ^2} \approx 2 +4 \, \left( \gamma -1 \right) ^2 \ \ \ ; \ \ \ \gamma \approx 1 \end{aligned}$$

and

37 $$\begin{aligned} -F(\gamma ) \approx -\frac{\pi }{2} + \frac{\pi }{8} \, \left( \gamma - 1 \right) ^2 \ \ \ ; \ \ \ \gamma \approx 1 . \end{aligned}$$

As a result, one can write:

38 $$\begin{aligned} \epsilon \left( \alpha =\frac{1}{\gamma },\gamma ,r_s \right) - \epsilon (\alpha =1,\gamma ,r_s) \approx \Bigg [ \frac{4}{r_s^2} \, \left( \gamma - 1 \right) ^2 -\frac{4}{3 \, \pi } \, \frac{1}{r_s} \, \left( \gamma - 1 \right) ^2 \Bigg ] \frac{k_e \, e^2}{2 \, a_B} \ \ \ ; \ \ \ \gamma \approx 1 . \end{aligned}$$

From here, one can calculate that $\Psi (\alpha =\frac{1}{\gamma })$ has lower energy than $\Psi (\alpha =1)$ for $r_{s} > 3 \, \pi$ as long as the anisotropy of the anisotropic Coulomb interaction potential is weak (for $\gamma \approx 1$ values).

At this juncture, it is useful to comment on how accurate is the prediction of a transition between an isotropic and anisotropic electron configuration at some critical value of the electron density. The HF approximation, as any variational method, may lead to artifacts like “phase transitions” when varying the external parameters. An example is the “phase transition” in the polaron theory, which is an artifact of the variational method^23,24^. However, there are equally valid many other cases in which the HF approximation provides qualitatively accurate predictions of a phase transition. For example, the HF approximation was the first to predict the transition from nonmagnetic to fully magnetic ground states in a free 3DEG as density decreases ($r_s$ increases). Of course, the precise value of the HF density at which the transition happens is not quantitatively accurate (neither should be expected to be so), but qualitatively speaking, the prediction of a spin polarization transition has been fully validated by extensive accurate QMC studies^4,25,26^. In the present case, the prediction of a transition between an isotropic and anisotropic electron configuration at some critical value of the electron density does not appear to be an artifact of the HF approximation. The lack of calculation of the correlation energy affects quantitatively the values of the energy for a given system of electrons as well as the value of the critical density at which the transition is predicted. However, we believe that the approximation will not change qualitatively the main conclusions drawn since we are interested in energy differences. In other words, energy differences between two electronic phases will likely be quite accurate (with or without the inclusion of the correlation energy) since the respective energies are calculated separately within the same approximation. Hence, the discrepancies/errors, which are systematic, are expected to be of the same order of magnitude and would tend to cancel out when energy differences are calculated. In a nutshell, the energies of the isotropic and anisotropic electronic phases are each separately affected by the nature of the approximation used. However, their energy differences (the one that matter to predict the transition) will likely be considerably more precise adding support to the comments stated above along the lines of substantiating the validity of this transition.

## Conclusions

In conclusion, we considered an anisotropic Coulomb interaction potential between particles that has never been considered before in the context of a 2DEG system. Such an anisotropic interaction potential depends on a phenomenological parameter denoted as $\gamma$. This choice represents a fundamentally novel element of the current work. Consideration of an anisotropic interaction potential between particles in a correlated system changes profoundly the properties of the system and may lead to unanticipated phenomena. For example, for the present $\gamma \ne 1$ case, we observe novel isotropic to anisotropic transitions between Fermi liquid phases (that never happen in a system with a standard isotropic Coulomb interaction potential).

The results of this work suggest many possible scenarios when it comes to an eventual elliptical deformation of the Fermi surface of a spinless 2DEG in presence of an anisotropic Coulomb interaction potential. Depending on the density of the system (value of $r_s$) the global energy mininum is somewhere between two special Fermi liquid states, $\Psi (\alpha =1)$ (the state with a circular Fermi surface that minimizes the kinetic energy) and $\Psi \left(\alpha =\frac{1}{\gamma }\right)$ (the state with an elliptically deformed Fermi surface in such a way as to mimimize the potential energy at any given value of $\gamma$).

For an anisotropic Coulomb interaction potential with $\gamma \ne 1$, the ground state is close to $\Psi (\alpha =1)$ at high density values of the system. For a small degree of anisotropy of the anisotropic Coulomb interaction potential, namely, for $\gamma \approx 1$, the state $\Psi \left(\alpha =\frac{1}{\gamma }\right)$ is energetically more favorable than $\Psi (\alpha =1)$ at lower densities that correspond to values of $r_s > 3 \, \pi$. However, this does not mean that $\Psi \left(\alpha =\frac{1}{\gamma }\right)$ is the lowest energy state of the system even at such low densities. In fact, the state with the lowest possible energy under general circumstances ($\gamma \ne 1$) is the one with an optimum value of the elliptical deformation parameter $\alpha$ somewhere between the values of $1/\gamma$ and 1. Such a state should be determined numerically from the minimization of the total energy of the system as a function of $\alpha$ for the given specific values of the parameters $r_s$ and $\gamma$. This means that the optimum elliptical deformation of the Fermi surface for the model adopted in this work is determined by a complex interplay of many factors including the density of the system.

To summarize, the results obtained in this work may provide useful insights on several rich scenarios that emerge when various anisotropic factors such as an anisotropic interaction potential, play a role on the stability of a 2DEG system. In particular, the ideas of this work may be tested experimentally in 2DEG systems confined in modulation-doped AlAs quantum wells or similar materials where there is an inherent piezo-electric effect of the samples. Since also the effective mass of the electrons is likely anisotropic in such systems one can experimentally tune at least one source of anisotropy (the interaction potential or the anisotropic effective mass) and look for experimental signatures of transport anisotropy in the specimens as a function of other quantities.

## Supplementary Information

Supplementary Information.

## Data Availability

The data are available upon request at ogciftja@pvamu.edu.

## References

[1] Pauli W (1925). On the connection of the arrangement of electron groups in atoms with the complex structure of spectra. Z. Phys..

[2] Dugdale SB (2016). Life on the edge: a beginner’s guide to the Fermi surface. Phys. Scr..

[3] Ceperley D (1978). Ground state of the fermion one-component plasma: a Monte Carlo study in two and three dimensions. Phys. Rev. B.

[4] Ceperley DM, Alder BJ (1980). Ground state of the electron gas by a stochastic method. Phys. Rev. Lett..

[5] Ceperley DM, Tanatar B (1989). Ground state of the two-dimensional electron gas. Phys. Rev. B.

[6] Kwon Y, Ceperley DM, Martin RM (1993). Effects of three-body and backflow correlations in the two-dimensional electron gas. Phys. Rev. B.

[7] Attaccalite C, Moroni S, Gori-Giorgi P, Bachelet GB (2002). Correlation energy and spin polarization in the 2D electron gas. Phys. Rev. Lett..

[8] Attaccalite C, Moroni S, Gori-Giorgi P, Bachelet GB (2003). Correlation energy and spin polarization in the 2D electron gas. Phys. Rev. Lett..

[9] Gori-Giorgi P, Moroni S, Bachelet GB (2004). Pair-distribution functions of the two-dimensional electron gas. Phys. Rev. B.

[10] Ciftja O (2019). Impact of an elliptical Fermi surface deformation on the energy of a spinless two-dimensional electron gas. Phys. Scr..

[11] Ciftja O, Livingston V, Thomas E (2017). Cyclotron motion of a charged particle with anisotropic mass. Am. J. Phys..

[12] Slater JC (1929). The theory of complex spectra. Phys. Rev..

[13] Wang H, Narayanan R, Wan X, Zhang F (2012). Fractional quantum Hall states in two-dimensional electron systems with anisotropic interactions. Phys. Rev. B.

[14] Ciftja O (2017). Anisotropic magnetoresistance and piezoelectric effect in GaAs Hall samples. Phys. Rev. B.

[15] De Poortere EP, Tutuc E, Shkolnikov YP, Vakili K, Shayegan M (2002). Magnetic-field-induced spin polarization of AlAs two-dimensional electrons. Phys. Rev. B.

[16] Ciftja O, Sutton B, Way A (2013). Energy in a finite two-dimensional spinless electron gas. AIP Adv..

[17] Ciftja O (2015). Hartree–Fock energy of a finite two-dimensional electron gas system in a jellium background. Physica B.

[18] Bernu B, Delyon F, Holzmann M, Baguet L, Bernu B, Delyon F, Holzmann M, Baguet L (2011). Hartree–Fock phase diagram of the two-dimensional electron gas. Phys. Rev. B.

[19] Bloch F (1929). Comment on the electron theory of ferromagnetism and electrical conductivity. Z. Phys..

[20] Rapisarda F, Senatore G (1996). Diffusion Monte Carlo study of electrons in two-dimensional layers. Aust. J. Phys..

[21] Ciftja O (2017). A result for the Coulomb electrostatic energy of a uniformly charged disk. Results Phys..

[22] Giuliani GF, Vignale G (2005). Quantum Theory of the Electron Liquid.

[23] Gerlach B, Löwen H (1991). Analytical properties of polaron systems or: do polaronic phase transitions exist or not?. Rev. Mod. Phys..

[24] Gerlach B, Smondyrev MA (2008). Upper and lower bounds for the large polaron dispersion in 1, 2, or 3 dimensions. Phys. Rev. B.

[25] Zong FH, Lin C, Ceperley DM (2002). Spin polarization of the low-density three-dimensional electron gas. Phys. Rev. E.

[26] Spink GG, Needs RJ, Drummond ND (2013). Quantum Monte Carlo study of the three-dimensional spin-polarized homogeneous electron gas. Phys. Rev. B.

