# Supplementary material for: Deformation of the Fermi surface of a spinless two-dimensional electron gas in presence of an anisotropic Coulomb interaction potential
Source: Sci Rep. 2021 Feb 4;11:3181. doi: 10.1038/s41598-021-82564-y (PMC7862404; doi:10.1038/s41598-021-82564-y)
Supplement: Supplementary file 1 — Supplementary Information. [file 41598_2021_82564_MOESM1_ESM.pdf]

APPENDICES A, B and C

Deformation of the Fermi surface of a spinless two-dimensional electron gas in presence of an anisotropic Coulomb interaction potential

Orion Ciftja

APPENDIX A: KINETIC ENERGY

We denote by $T(\alpha)$ the total kinetic energy of the system, namely, the expectation value of the total kinetic energy operator with respect to the wave function $\Psi(\alpha)$ parametrized by the elliptical surface deformation parameter α defined in Eq.(12). In the thermodynamic limit, one transforms the sum over $\vec{k}$ into an integral over $\vec{k}$ and obtains:

$$T(\alpha) = \frac{A}{(2\pi)^2} \frac{\hbar^2}{2\,m} \iint\limits_{D_{\vec{k}}:\left\{\frac{k_x^2}{k_a^2}+\frac{k_y^2}{k_b^2}\leq 1\right\}} dk_x\, dk_y \, \left(k_x^2 + k_y^2\right) \ , \tag{A1}$$

where $D_{\vec{k}}$ is the region bounded by an ellipse in $\vec{k}$-space. The integral above is simple and the final result is written as:

$$T(\alpha) = N \frac{\hbar^2}{2\,m} \frac{\left(k_a^2 + k_b^2\right)}{4} \ , \tag{A2}$$

where we used the fact that the total number of spinless electrons is:

$$N = \frac{A}{(2\pi)^2} \pi\, k_a\, k_b \ . \tag{A3}$$

Based on the definition of parameter α from Eq.(12), one writes the kinetic energy per particle as:

$$t(\alpha) = \frac{T(\alpha)}{N} = \frac{\epsilon_F}{4} \left(\alpha^2 + \frac{1}{\alpha^2}\right) \ , \tag{A4}$$

where

$$\epsilon_F = \frac{\hbar^2\, k_F^2}{2\,m} \ , \tag{A5}$$

is the Fermi energy for a circular Fermi surface.

APPENDIX B: ONE-PARTICLE DENSITY MATRIX

By transforming the sum over $\vec{k}$ into an integral over $\vec{k}$ one can write the one-particle density matrix in the thermodynamic limit as:

$$\rho(\alpha, \vec{r}_1, \vec{r}_2) = \frac{1}{(2\pi)^2} \iint\limits_{D_{\vec{k}}:\left\{\frac{k_x^2}{k_a^2}+\frac{k_y^2}{k_b^2}\leq 1\right\}} dk_x\, dk_y\, e^{i\,\vec{k}\,(\vec{r}_2-\vec{r}_1)} \ , \tag{B1}$$

where α is defined in Eq.(12) and $D_{\vec{k}}$ is the region bounded by the ellipse in $\vec{k}$-space. The integration can be done analytically and the final result is:

$$\rho(\alpha, \vec{r}_1, \vec{r}_2) = 2\,\rho_0\, \frac{J_1\left(k_F\sqrt{\alpha^2\, x_{21}^2 + \frac{y_{21}^2}{\alpha^2}}\right)}{k_F\sqrt{\alpha^2\, x_{21}^2 + \frac{y_{21}^2}{\alpha^2}}} \ , \tag{B2}$$

where $\rho_0 = k_a k_b/(4\pi) = k_F^2/(4\pi)$ is the uniform density of the system, $J_1(x)$ is a Bessel function of the first kind and $\vec{r}_{21} = \vec{r}_2 - \vec{r}_1 = (x_{21}, y_{21})$ is a separation vector. One has $\alpha = 1$ for a circular Fermi surface which leads to the following expression for the one-particle density matrix:

$$\rho(\alpha = 1, \vec{r}_1, \vec{r}_2) = 2\,\rho_0\,\frac{J_1\left(k_F\,r_{21}\right)}{k_F\,r_{21}}\ , \tag{B3}$$

where $r_{21} = \sqrt{x_{21}^2 + y_{21}^2}$ is the radial separation distance.

APPENDIX C: POTENTIAL ENERGY

In order to calculate the total potential energy in Eq.(15) we use the expression for $\rho(\alpha, \vec{r}_1, \vec{r}_2)$ given from Eq.(B2) and write the total potential energy as:

$$U(\alpha,\gamma) = -2\,\rho_0^2 \int_\Omega d^2r_1 \int_\Omega d^2r_2 \left[\frac{J_1\left(k_F\sqrt{\alpha^2\,x_{21}^2 + \frac{y_{21}^2}{\alpha^2}}\right)}{k_F\sqrt{\alpha^2\,x_{21}^2 + \frac{y_{21}^2}{\alpha^2}}}\right]^2 v_\gamma(x_{21}, y_{21})\ , \tag{C1}$$

where

$$v_\gamma(x_{21}, y_{21}) = v_\gamma(\vec{r}_{21}) = v_\gamma(\vec{r}_2 - \vec{r}_1) = \frac{k_e\,e^2}{\sqrt{\frac{x_{21}^2}{\gamma^2} + \gamma^2\,y_{21}^2}}\ . \tag{C2}$$

The potential energy per particle in the thermodynamic limit can be written as:

$$u(\alpha,\gamma) = \frac{U(\alpha,\gamma)}{N} = -2\,\rho_0 \int_{\Omega\to\infty} d^2r_{21} \left[\frac{J_1\left(k_F\sqrt{\alpha^2\,x_{21}^2 + \frac{y_{21}^2}{\alpha^2}}\right)}{k_F\sqrt{\alpha^2\,x_{21}^2 + \frac{y_{21}^2}{\alpha^2}}}\right]^2 v_\gamma(x_{21}, y_{21})\ . \tag{C3}$$

In the thermodynamic limit, $L \to \infty$ which means that $-\infty < x_{21}, y_{21} < +\infty$. Therefore, the quantity to calculate is:

$$u(\alpha,\gamma) = -2\,\rho_0 \int_{-\infty}^{+\infty} dx_{21} \int_{-\infty}^{+\infty} dy_{21} \left[\frac{J_1\left(k_F\sqrt{\alpha^2\,x_{21}^2 + \frac{y_{21}^2}{\alpha^2}}\right)}{k_F\sqrt{\alpha^2\,x_{21}^2 + \frac{y_{21}^2}{\alpha^2}}}\right]^2 \frac{k_e\,e^2}{\sqrt{\frac{x_{21}^2}{\gamma^2} + \gamma^2\,y_{21}^2}}\ , \tag{C4}$$

where we substituted the form of $v_\gamma(x_{21}, y_{21})$ from Eq.(C2). We now introduce new auxiliary variables that make the one-particle density matrix "isotropic", namely, we introduce the following new variables defined as:

$$x' = \alpha\,x_{21}\quad ;\quad y' = \frac{y_{21}}{\alpha}\quad ;\quad dx'dy' = dx_{21}\,dy_{21}\ . \tag{C5}$$

With this transformation the quantity in Eq.(C4) can be rewritten as:

$$u(\alpha,\gamma) = -2\,\rho_0\,k_e\,e^2 \int_{-\infty}^{+\infty} dx' \int_{-\infty}^{+\infty} dy' \left[\frac{J_1\left(k_F\sqrt{x'^2 + y'^2}\right)}{k_F\sqrt{x'^2 + y'^2}}\right]^2 \frac{1}{\sqrt{\frac{x'^2}{\alpha^2\,\gamma^2} + \alpha^2\,\gamma^2\,y'^2}}\ . \tag{C6}$$

Now we change to a 2D polar system of coordinates:

$$x' = r'\cos(\varphi')\quad ;\quad y' = r'\sin(\varphi')\ . \tag{C7}$$

This additional change of variables allows us to write the potential energy per particle as:

$$u(\alpha,\gamma) = -2\,\rho_0\,k_e\,e^2 \int_0^\infty dr' \left[\frac{J_1\,(k_F\,r')}{(k_F\,r')}\right]^2 \int_0^{2\pi} d\varphi' \frac{1}{\sqrt{\frac{\cos^2(\varphi')}{\alpha^2\gamma^2} + \alpha^2\gamma^2\sin^2(\varphi')}} \;. \tag{C8}$$

The integration over the radial variable can be done with the following integral formula:

$$\int_0^\infty dx \left[\frac{J_1(x)}{x}\right]^2 = \frac{4}{3\,\pi} \;. \tag{C9}$$

The integration over the angular variable can be done with the following integral formula:

$$\int_0^{2\pi} \frac{d\varphi}{\sqrt{a^2\,\cos^2(\varphi) + b^2\,\sin^2(\varphi)}} = \frac{4}{b}\,K\left(m = 1 - \frac{a^2}{b^2}\right) \;, \tag{C10}$$

where $K(m)$ is a complete elliptic integral of the first kind with parameter, m defined as

$$K(m) = \int_0^{\pi/2} \frac{d\,\theta}{\sqrt{1 - m\,\sin^2(\theta)}} \;. \tag{C11}$$

By using the formula in Eq.(C10) it is easy to prove that:

$$\int_0^{2\pi} d\varphi' \frac{1}{\sqrt{\frac{\cos^2(\varphi')}{\alpha^2\gamma^2} + \alpha^2\gamma^2\sin^2(\varphi')}} = \frac{4}{\alpha\,\gamma}\,K\left(m = 1 - \frac{1}{\alpha^4\,\gamma^4}\right) \;, \tag{C12}$$

Let's define an auxiliary function $F(x)$ written as:

$$F(x) = \frac{1}{x} K\left(m = 1 - \frac{1}{x^4}\right) \;. \tag{C13}$$

After some simple algebraic manipulations and relying on the fact that $\rho_0 = k_F^2/(4\pi)$ we can write the potential energy per particle as:

$$u(\alpha,\gamma) = -\frac{8}{3\pi^2} k_F\,k_e\,e^2\,F(\alpha\,\gamma) \;, \tag{C14}$$

where $F(x)$ is the auxiliary function in Eq.(C13). Note that the argument of $F(x)$ in Eq.(C14) is the product of parameters α and γ.
